# Using a 3D Virtual Supermarket to Measure Food Purchase Behavior: A Validation Study

**DOI:** 10.2196/jmir.3774

**Published:** 2015-04-28

**Authors:** Wilma Elzeline Waterlander, Yannan Jiang, Ingrid Hendrika Margaretha Steenhuis, Cliona Ni Mhurchu

**Affiliations:** ^1^National Institute for Health InnovationSchool of Population HealthUniversity of AucklandAucklandNew Zealand; ^2^Department of Health Sciences and the EMGO Institute for Health and Care ResearchFaculty of Earth and Life SciencesVU University AmsterdamAmsterdamNetherlands

**Keywords:** virtual reality, user-computer interface, software validation, nutrition policy, food, behavior, public health

## Abstract

**Background:**

There is increasing recognition that supermarkets are an important environment for health-promoting interventions such as fiscal food policies or front-of-pack nutrition labeling. However, due to the complexities of undertaking such research in the real world, well-designed randomized controlled trials on these kinds of interventions are lacking. The Virtual Supermarket is a 3-dimensional computerized research environment designed to enable experimental studies in a supermarket setting without the complexity or costs normally associated with undertaking such research.

**Objective:**

The primary objective was to validate the Virtual Supermarket by comparing virtual and real-life food purchasing behavior. A secondary objective was to obtain participant feedback on perceived sense of “presence” (the subjective experience of being in one place or environment even if physically located in another) in the Virtual Supermarket.

**Methods:**

Eligible main household shoppers (New Zealand adults aged ≥18 years) were asked to conduct 3 shopping occasions in the Virtual Supermarket over 3 consecutive weeks, complete the validated Presence Questionnaire Items Stems, and collect their real supermarket grocery till receipts for that same period. Proportional expenditure (NZ$) and the proportion of products purchased over 18 major food groups were compared between the virtual and real supermarkets. Data were analyzed using repeated measures mixed models.

**Results:**

A total of 123 participants consented to take part in the study. In total, 69.9% (86/123) completed 1 shop in the Virtual Supermarket, 64.2% (79/123) completed 2 shops, 60.2% (74/123) completed 3 shops, and 48.8% (60/123) returned their real supermarket till receipts. The 4 food groups with the highest relative expenditures were the same for the virtual and real supermarkets: fresh fruit and vegetables (virtual estimate: 14.3%; real: 17.4%), bread and bakery (virtual: 10.0%; real: 8.2%), dairy (virtual: 19.1%; real: 12.6%), and meat and fish (virtual: 16.5%; real: 16.8%). Significant differences in proportional expenditures were observed for 6 food groups, with largest differences (virtual – real) for dairy (in expenditure 6.5%, *P*<.001; in items 2.2%, *P*=.04) and fresh fruit and vegetables (in expenditure: –3.1%, *P*=.04; in items: 5.9%, *P*=.002). There was no trend of overspending in the Virtual Supermarket and participants experienced a medium-to-high presence (88%, 73/83 scored medium; 8%, 7/83 scored high).

**Conclusions:**

Shopping patterns in the Virtual Supermarket were comparable to those in real life. Overall, the Virtual Supermarket is a valid tool to measure food purchasing behavior. Nevertheless, it is important to improve the functionality of some food categories, in particular fruit and vegetables and dairy. The results of this validation will assist in making further improvements to the software and with optimization of the internal and external validity of this innovative methodology.

## Introduction

Supportive environments are essential for people to make healthier food choices and there is a growing call to implement structural interventions such as fiscal policies and front-of-pack labeling to create a healthier food environment [1-3]. Supermarkets form a key setting for health-promoting interventions [4,5] because they have a central position within the food chain [6,7] and they are where people buy most of their food (87% of households in New Zealand buy foods from supermarkets weekly or more often [8]). Although structural interventions in the retail setting have clear potential, high-quality evidence on their effectiveness is limited [9,10]. Evidence from randomized controlled trials (RCTs) is particularly lacking. Although examples of high-quality supermarket trials exist [10-12], experimental studies have mostly been conducted in settings with a limited number of food choices and most lacked objective outcome measures [13].

The main reason for the dearth of high-quality supermarket trials is that they are complex and costly to conduct. This is especially true for RCTs (eg, it would be problematic to raise prices on soft drinks [simulating a tax] in some supermarkets but not in others). Furthermore, opposition and extensive lobbying from the food industry against several proposed intervention strategies is another important reason for the lack of experimental studies [14].

**Figure 1:**
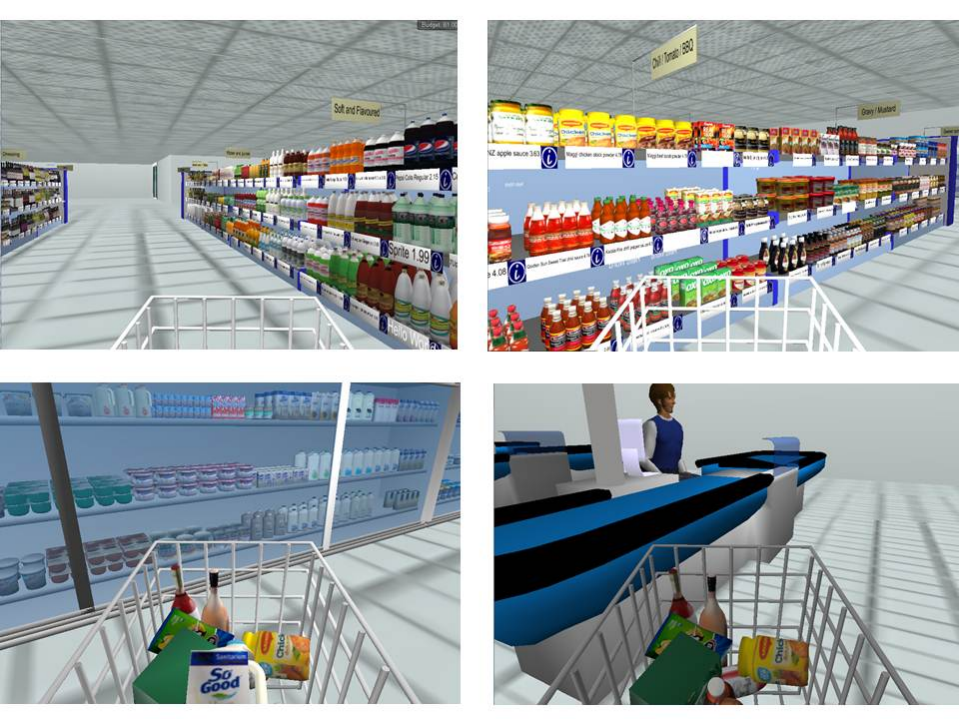
Screenshots of the New Zealand Virtual Supermarket.

In order to find a solution to the complexities of undertaking supermarket intervention research, we developed a research tool that can be used to study the effects of interventions in a virtual reality setting: the Virtual Supermarket (see Figure 1). Virtual environments are computer-generated models, which participants can experience and interact with intuitively in real time [15]. Gaming technology is used to simulate a real supermarket shopping experience where study participants purchase virtual food items. Photographs of real foods are used to compose virtual food products and prices are displayed on virtual shelf labels. Currently, 2 versions of this Virtual Supermarket exist: the original Dutch version [16] and a New Zealand version (developed using a similar methodology) that is the subject of this study. Other examples of virtual supermarkets can be found in the scientific literature; however, to our best knowledge, these have different functionalities [17], are developed for other purposes (eg, rehabilitation) [18], or have not published study results yet [19].

To obtain valid study results, it is important that the Virtual Supermarket simulates a real supermarket as closely as possible. Here, one of the most important traits is the level of presence people experience [15]. *Presence* is defined as “a psychological state of ‘being there’ mediated by an environment that engages our senses, captures our attention, and fosters our active involvement” [20] and reflects “the subjective experience of being in one place or environment, even if one is physically located in another” [21]. Factors that are hypothesized to contribute to a sense of presence are (1) involvement, (2) sensory fidelity, (3) adaptation/immersion, and (4) interface quality factors. Second, a key question is whether peoples’ virtual behavior reflects their real-life behavior accurately. Studies using the Dutch Virtual Supermarket have already shown some promising results [22,23]. However, to date, validation of the software has been solely based on self-report (questionnaire) data. Therefore, the aim of this study was to validate the NZ Virtual Supermarket by comparing virtual and real-life food purchasing behavior using objective outcome measures and also to evaluate the level of presence that people experience. Results will inform further development of the software and guide the development of future experiments using the Virtual Supermarket.

## Methods

### The New Zealand Virtual Supermarket

The Virtual Supermarket was developed in Unity (a game development system) and represents a 3-dimensional (3D) computer simulation of a real shopping experience (Figure 1). More details about the software can be found elsewhere [16,24]. The Dutch Virtual Supermarket is available online [25] (the NZ version is currently undergoing modification).

The NZ Virtual Supermarket was designed using the Auckland branch of a market leader as a model. NZ food prices and product range were obtained from a 2011 field survey of packaged foods available for sale in Auckland supermarkets [26] and online supermarkets. Foods were categorized into 18 food groups and further divided into 91 categories, 141 subcategories, and 31 sub-subcategories (eg, dairy—cheese—hard cheese—high salt cheese). The Virtual Supermarket includes a representative number of products in each food category, with at least 1 product in each subcategory, adding up to a total of 1445 unique products (Table 1) (an average NZ supermarket offers approximately 9000 nutritionally unique food products). Within each category, we selected the top-selling products using the Australian Grocery Guide annual report 2010 sales data [27]. In addition, we ensured sufficient variety of special food items such as organic, diet, or gluten-free varieties and included home or discount brands where possible.

In the Virtual Supermarket, participants can navigate around the store with a shopping trolley using their computer cursor keys and select (or remove) groceries with a mouse click. There is an option to view a list of selected groceries, including the price and total amount of money spent. Cash registers are located at the end of the supermarket where participants can check out and complete the study. The software underwent some testing by our study team prior to this validation study, but no formal usability testing was performed.

**Table 1:** Food groups and food categories within the New Zealand Virtual Supermarket.

Food group and category		Products, n
**Alcoholic beverages**		
	Wine (including cask wine, champagne, dessert wine, red, rose wine, sparkling, special releases, white wine, other liquor)	57
	Beer and cider	15
**Baby care**		
	Baby food	13
	Baby formula	4
**Bread and bakery**		
	Rolls, bagels, and flat breads	21
	Fresh bread (in-store bakery)	4
	Sweet treats and cakes	28
	Biscuits	73
	Bread (packaged)	43
	Cakes, muffins, and pastry	26
**Baking and cooking**		
	Herbs and spices	40
	Baking additives	12
	Bread crumbs and coating	4
**Beverages** (including coffee, plain; cordial bases; electrolyte drink; energy drink; fruit and vegetable juices; hot drink mixes; ice tea drinks; soft and flavored drinks; tea, plain; water)		115
**Cereal and cereal products**		
	Bran and breakfast cereals	27
	Cereal bars	23
	Flour	4
	Grains	4
	Noodles	5
	Pasta	31
	Rice	15
	Sugar	4
**Convenience foods**		
	Pizza	5
	Ready meals	23
	Ready snacks	10
	Soup	18
**Dairy**		
	Cheese	62
	Cream	5
	Desserts	7
	Ice cream	20
	Milk	22
	Yogurt/ yogurt drinks	20
**Deli and chilled foods**		
	Fresh pasta, sauces, soups, and pesto	4
	Antipasto and continental meats	12
	Fresh meals and sandwiches	3
	Chilled salads	3
	Sliced meats	9
**Edible oils and oil emulsions**		
	Butter and margarine	11
	Cooking oil/spray	9
**Eggs**		
	Free range	2
	Cage eggs	2
	Barn laid	1
**Fish and fish products**		
	Canned fish	19
	Frozen fish	14
	Chilled fish	5
	Fish with pastry	1
**Fresh fruit and vegetables**		
	Fruit	27
	Fresh herbs	7
	Green vegetables	7
	Prepared salads	5
	Other vegetables	22
**Fruit and vegetables other**		
	Fruit (including dried, dried fruit and nut mixes, frozen fruit, fruit bars, fruit in juice/syrup, other)	40
	Vegetables (including baked beans, corn [can], legumes [can], tomatoes [can], canned [other], dried legumes, frozen potato products, frozen vegetables)	61
	Jam and spreads	11
	Nuts and seeds	22
	Pickled vegetables	11
**Fresh meat and seafood, meat products**		
	Fresh fish	5
	Shell fish	3
	Smoked fish	1
	Beef and veal (unprocessed)	5
	Lamb (unprocessed)	4
	Pork (unprocessed)	4
	Poultry (unprocessed)	2
	Sausages and small good	5
	Meat alternatives	6
	Processed meat	63
**Other miscellaneous**		
	Other desserts (including frozen juice and ice blocks, fruit crumbles, sponges/pudding, fruit pies, jelly, pavlova/meringues, sorbets, other desserts)	21
**Sauces, spreads and seasonings**		
	Mayonnaise/dressings	15
	Meal accompaniments	1
	Sauces/seasonings	95
	Spreads	23
	Other savory	1
	Pate	6
	Peanut butter and sweet spreads	9
	Relished and pickles	12
	Yeast extract	2
**Snack foods**		
	Crisps and snacks	32
	Sweet snacks	54
	Chewing gum	7
Total		1445

### Study Design

Study participants were asked to shop on 3 occasions in the Virtual Supermarket over 3 consecutive weeks and to collect their real supermarket grocery till receipts for that same period. Virtual and real shops were conducted in the same weeks. Three shopping occasions were included to provide a representative picture of usual food purchases and to test learning effects. Participants were instructed to conduct their weekly virtual shop prior to their real-life shopping trips. This order was chosen because it is unlikely that people adapt their real-life purchases to their choices made in the Virtual Supermarket; however, when real shops are conducted first, it is possible that people could use their real till receipts as a basis for their food purchases in the Virtual Supermarket. The study design and procedures were approved by the University of Auckland Human Participants Ethics Committee (Nov 7, 2012; ID8691).

### Participants

Participants were recruited from the general NZ population using advertisements placed in supermarkets, a research participant recruitment website (researchstudies.co.nz), local newspapers, mailing lists and via Māori (indigenous New Zealanders) and Pacific networks within the University. Inclusion criteria were adult (≥18 years), main household shopper, able to communicate in English, access to a computer and Internet, and have an email address. Exclusion criteria included another person in the household already participating in the study.

### Study Procedure

Participants completed the study at home or on any computer with an Internet connection. Following registration and consent, an email was sent with study instructions and a URL (link to Web address) to download the Virtual Supermarket. In addition to a hard copy illustrated manual, we recorded an instructional video on the Virtual Supermarket. For all 3 virtual shopping occasions, participants were instructed to purchase food for their household for the coming week as they planned to do in real life. It was emphasized that they should buy *all* food groceries they would need for their household in the coming week, including food they were planning to buy for a special occasion (eg, for a birthday). Likewise, participants were instructed not to buy foods in the Virtual Supermarket if they were not planning to buy these in real life (ie, because they had a sufficient stock). The shopping budget in the Virtual Supermarket was set to the same level as participants’ real-life household food shopping budget for that week. After completion of each virtual shop, participants were asked to collect their real supermarket till receipts for that week (a reminder message appeared on their computer screen). Participants were instructed to collect all real-life food shop till receipts, including from supermarkets, speciality stores, convenience stores, and markets, but not from fast food, restaurants, or takeaways. Along with the till receipts, participants completed a questionnaire about their shopping habits that week (eg, “In the past week, did you make any special food purchases that you would not have normally; for example, because there was a sale/promotion or because of a special occasion?”) and asking how many till receipts were missing. Participants received email and short message service (SMS) text message reminders throughout the study to remind them to complete their virtual shops and collect their till receipts. Participants who completed all 3 virtual shops and returned their grocery till receipts received a NZ $20 supermarket voucher.

### Outcome Measures

#### Primary Outcome

The primary outcome of this study was the proportional expenditures (NZ$) on each food group (18 food groups, see Table 1) for both the Virtual Supermarket and real supermarket (eg, percentage of total expenditures on vegetables). We measured proportional expenditure instead of absolute expenditures because we were interested in shopping patterns (eg, did participants buy similar quantities of milk and bread) which are better reflected by proportional expenditures (absolute supermarket prices tend to vary substantially over time). Secondly, we also looked at the number of products purchased in each food category and compared the Virtual Supermarket and real supermarket proportions (eg, percentage of all purchased products that are vegetables). Product purchases in the Virtual Supermarket were measured by till receipts automatically generated at the end of each shop. Real-life food purchases were measured by grocery till receipts.

#### Secondary Outcomes

Secondary outcomes included self-reported level of presence measured by the Presence Questionnaire Items Stems (version 4.0) [20]. This questionnaire includes a total of 29 items that are scored on a 7-point Likert scale. Examples of questions include “How responsive was the Virtual Supermarket to actions that you initiated (or performed)?” and “How much did your experiences in the Virtual Supermarket seem consistent with your real world experiences?” In addition, we measured potential effect modifiers and background variables including:

1. Capability or tendency to be involved or immersed. People can experience different levels of involvement both in virtual tasks and in common activities (focus). This concept was measured with the items that relate to the factor “focus” (7 questions) from the Immersive Tendency Questionnaire Item Stems [21] (ie, “how mentally alert do you feel at the present time?”)
2. Participant characteristics including age, gender, household size, ethnicity, responsibility for household grocery shopping, highest educational qualification, work status, household income, supermarket shopping habits, computer use, and Internet access.

### Statistical Analyses

#### Sample Size

We aimed to recruit 96 participants. This sample size was estimated to provide 85% power at 5% level of significance (2-sided) to detect a minimal absolute difference of 1% in percentage of food products or expenditure on each food category between weekly virtual and real supermarket purchases. This assumed a standard deviation of 3% based on data from previous research [23] and an estimated 20% loss to follow-up rate. Because this was a validation study, the sample size was not adjusted for multiple comparisons.

#### Comparing Virtual and Real Supermarkets

Data from the virtual and real-life grocery till receipts were entered into SPSS version 20 (IBM Corp, Armonk, NY, USA) and coded within the matching 18 food groups. Data were then imported into SAS version 9.2 (SAS Institute Inc, Cary, NC, USA) for further analysis. For each weekly shopping visit, proportional expenditure (NZ$) and the proportion of products purchased were calculated for each food group. First, alignment of the virtual and the real purchases was described using line graphs displaying mean purchases for all food groups for all participants with a least 1 valid virtual shop (raw data). Second, the resemblance between the virtual and real supermarket was tested using repeated measures mixed models. Both supermarket type and shopping visit were fitted as fixed effects. Their interaction was tested and dropped if it was not statistically significant. A random effect at participant level was also fitted to account for correlation between repeated visits on the same participant, and attrition over time assuming the data were missing at random. Model-adjusted means were estimated for the virtual and real supermarkets, and mean differences were tested. Analyses were conducted on the total sample (ie, including all participants who completed at least 1 virtual shop, N=86), followed by complete-case analysis. This second phase included participants who completed all 3 virtual shops and returned grocery till receipts for all 3 weeks. Also, the till receipts had to be valid and we excluded those who reported having failed to return the majority of their receipts (n=8). To determine missing till receipts, we asked about the number of shops participants conducted during the week, whether these were small or bigger shopping episodes, and how many till receipts they collected. If participants reported having conducted a large shop, this till receipt had to be available for the participant’s data to be included in analysis. If participants did multiple smaller shops, at least 60% of receipts had to be available. This resulted in a total of 52 participants (60%, 52/86 for the complete-case analysis).

#### Questionnaire Data

After each virtual shopping occasion, participants were asked to complete the presence and focus questionnaires. Because there was no difference in these scores over time, the mean total presence and focus score over all completed shops was derived for each participant (typical value). These mean values were divided into 3 groups (low/medium/high) for descriptive analysis. Linear mixed models were used to test the predicted effect of focus on presence (continuous score). Sensitivity analysis was conducted to test whether participants with a higher presence score performed better in the Virtual Supermarket within the complete cases by selecting a subset of participants with a high sense of presence using the median score (median 100) as a cut-off point.

## Results

### Participant Characteristics

**Figure 2:**
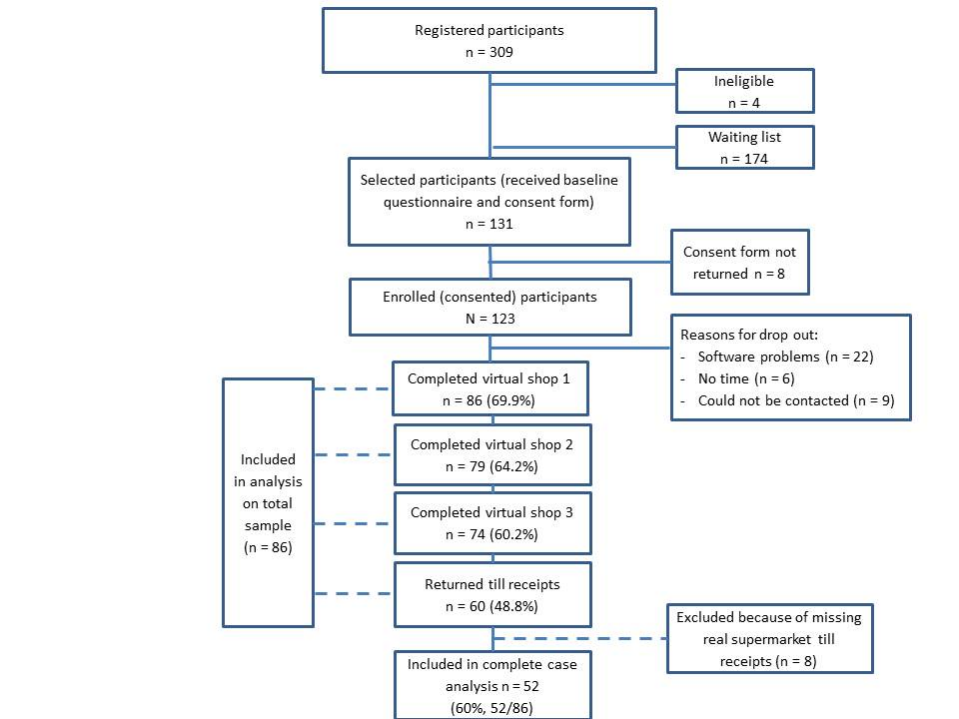
Study flow diagram.

A total of 309 participants registered for this study via phone or email. The first 135 participants were phoned to check their eligibility; 4 were not eligible because they were unavailable during the study period. The remainder were sent the baseline questionnaire and consent forms, with 123 participants enrolled in the study. Of these, 86 (69.9%, 86/123) participants completed shop 1, 79 (64.2%, 79/123) completed shop 2, 74 (60.2%, 74/123) completed shop 3, and 60 (48.8%, 60/123) returned their till receipts (see Figure 2).

Table 2 shows the characteristics of the participants who completed at least 1 virtual shop (N=86), completed all 3 virtual shops and returned valid till receipts (N=52), and completed all 3 virtual shops and returned valid till receipts plus had a high presence score (N=25). Results show that there were no clear differences in these sets of participants, except that participants in group 3 (high presence) were somewhat younger (mean 34.0, SD 8.5 years compared to mean 38.7, SD 12.3 years for all 86 participants), and included fewer retired persons and more students.

**Table 2:** Participant characteristics (N=86).

Participant characteristics		All participants^a^ N=86	Complete cases^b^ n=52	High presence^c^ n=25
Age (years), mean (SD)		38.7 (12.3)	38.0 (11.2)	34.0 (8.5)
Gender (female), n (%)		66 (76.7)	41 (79)	20 (80)
**Household size, n (%)**				
	1 person	9 (10.5)	7 (14)	3 (12)
	2 persons	32 (37.2)	19 (37)	10 (40)
	3 persons	15 (17.4)	9 (17)	6 (24)
	≥4 persons	28 (32.6)	16 (31)	6 (16)
	Missing	2 (2.3)	1 (2)	—
**Ethnicity, n (%)**				
	NZ European	45 (52.3)	25 (48)	12 (48)
	Māori	3 (3.5)	2 (4)	1 (4)
	Pacific Islander	3 (3.5)	1 (2)	1 (4)
	Chinese	6 (7.0)	4 (8)	1 (4)
	Other (Australian, English, etc)	29 (33.7)	20 (38)	10 (36)
**Responsibility for household grocery shopping, n (%)**				
	Mainly responsible	51 (59.3)	32 (62)	14 (56)
	Mostly responsible	17 (19.8)	7 (14)	3 (12)
	Shared responsibility	17 (19.8)	13 (25)	8 (32)
	Missing	1 (1.2)	—	—
**Highest educational qualification, n (%)**				
	None	4 (4.7)	2 (4)	—
	Secondary school	11 (12.8)	5 (10)	3 (12)
	Undergraduate	26 (30.2)	19 (37)	9 (36)
	Postgraduate	34 (39.5)	20 (39)	11 (44)
	Other	4 (4.7)	3 (6)	1 (4)
	Declined answer	7 (8.2)	3 (6)	1 (4)
**Work status, n (%)**				
	Full time paid work	34 (39.5)	22 (42)	12 (48)
	Part time paid work	14 (16.3)	9 (17)	4 (16)
	Retired	6 (7.0)	3 (6)	0 (0)
	Student	11 (12.8)	6 (12)	4 (16)
	Homemaker	11 (12.8)	7 (14)	4 (16)
	Beneficiary	2 (2.3)	0 (0)	0 (0)
	Other	7 (8.2)	5 (10)	1 (4)
	Missing	1 (1.2)	—	—
**Total household income before tax** ^d^ **(NZ $), n (%)**				
	<$20,000	5 (5.8)	4 (8)	2 (8)
	$20,001-$40,000	11 (12.8)	7 (13)	3 (12)
	$40,001-$60,000	11 (12.8)	5 (10)	1 (4)
	$60,001-$80,000	16 (18.6)	9 (17)	7 (28)
	≥$80,001	39 (45.4)	20 (39)	11 (44)
	Don’t know/declined answer	4 (4.7)	2 (4)	1 (4)
**Food purchases at supermarket, n (%)**				
	All food	9 (10.5)	6 (12)	4 (16)
	Most food	62 (72.1)	36 (70)	17 (68)
	Half of food	12 (14.0)	9 (17)	3 (12)
	Some food	2 (2.4)	1 (2)	1 (4)
	Missing	1 (1.2)	—	—
**Grocery shopping frequency, n (%)**				
	< Once a week	10 (11.6)	5 (10)	2 (8)
	Once a week	31 (36.0)	16 (31)	8 (32)
	Twice a week	29 (33.7)	22 (42)	10 (40)
	≥3 times a week	15 (17.5)	9 (17)	5 (20)
	Missing	1 (1.2)	—	—
**Frequency of using a computer, n (%)**				
	Daily	83 (96.5)	51 (98)	24 (96)
	Several days per week	2 (2.3)	1 (2)	1 (4)
	Missing	1 (1.2)	—	—
Internet at home (yes), n (%)		85 (98.8)	51 (98)	24 (96)
**Type of operating system used for this study, n (%)**				
	Windows XP	11 (12.8)	6 (12)	4 (16)
	Windows 7	41 (47.7)	26 (50)	11 (44)
	Windows (don’t know version)	20 (23.3)	11 (21)	7 (28)
	MacOS X 10.5/10.6	5 (5.8)	3 (6)	1 (4)
	MacOS X 10.7/10.8	6 (7.0)	5 (10)	1 (4)
	MacOS X (don’t know version)	2 (2.3)	1 (2)	1 (4)
	Missing	1 (1.2)	—	—

^a.^ Includes all participants who completed at least 1 shop in the Virtual Supermarket.

^b.^ Includes all participants who completed all 3 shops in the Virtual Supermarket and returned valid till receipts.

^c.^ Includes participants who completed all 3 shops in the Virtual Supermarket and returned valid till receipts and had a high presence score.

^d.^ Median annual income in New Zealand from wages and salaries was NZ $43,888 in June 2013 (per person)

### Descriptive Analysis

**Figure 3:**
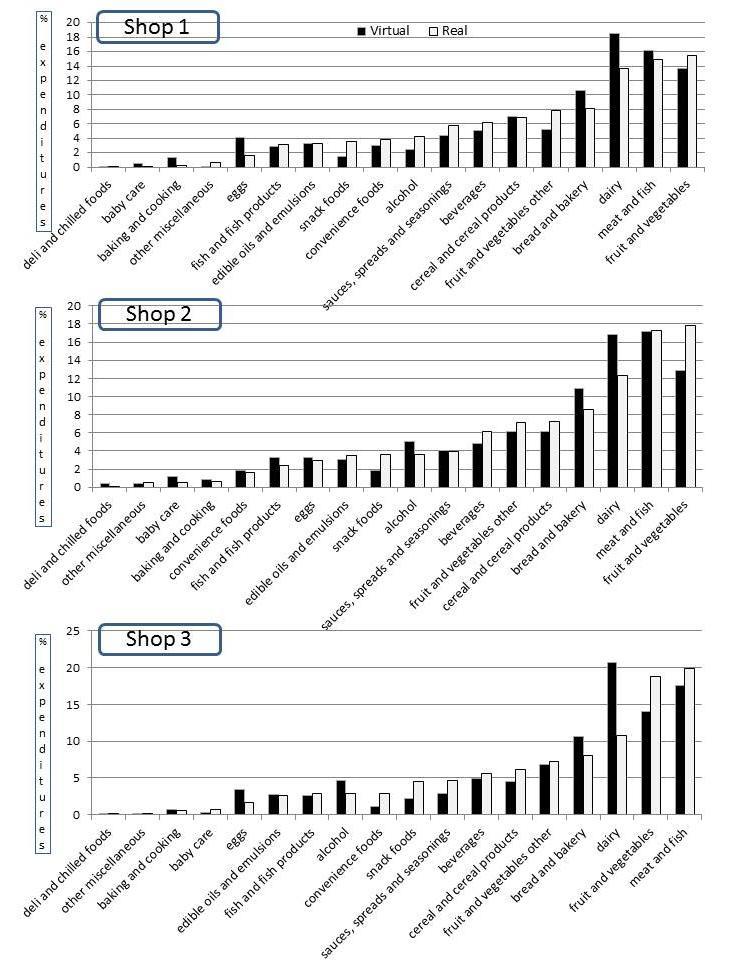
Proportion (%) expenditures for the 18 foods groups for the virtual and real shopping occasions (raw data) (n=86).

**Figure 4:**
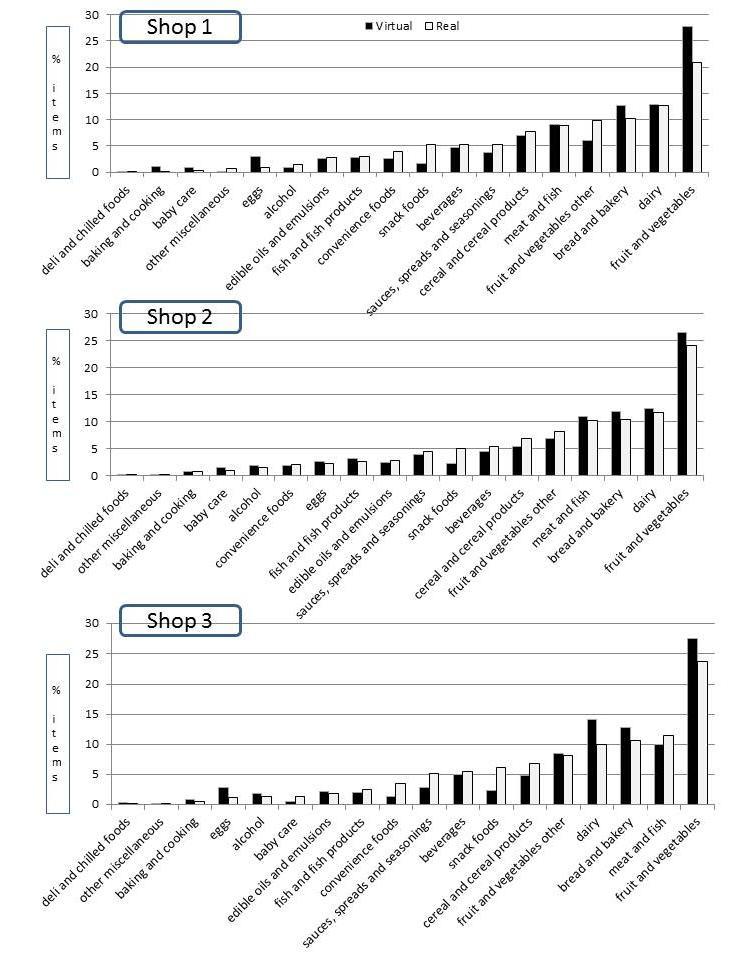
Proportion (%) of items purchased for the 18 foods groups for the virtual and real shopping occasions (raw data) (n=86).

Figures 3 and 4 show the proportion of money spent / proportion of items purchased for each of the 18 food groups, respectively, for both the virtual and the real supermarket and for each of the 3 shopping occasions (n=86). Results suggest that the purchasing patterns across the food groups were comparable. The 4 food groups with the highest relative expenditures were the same for the virtual and real supermarkets (all 3 shops): fruit and vegetables (14.3% Virtual Supermarket and 17.4% real supermarket), bread and bakery (10.0% Virtual Supermarket and 8.2% real supermarket), dairy (19.1% Virtual Supermarket and 12.6% real supermarket), and meat and fish (16.5% Virtual Supermarket and 16.8% real supermarket) (Table 3). Overall, total mean expenditures in the Virtual Supermarket were NZ $84.96 (SD 46.88 compared to NZ $125.15 (SD 74.15) in the real supermarket. On average, participants set their budget in the Virtual Supermarket at NZ $121.19 (SD 65.01) and spent 71.4% (SD 25.6) of this budget.

**Table 3:** Means and differences for Virtual Supermarket and real supermarket purchases for the 18 food categories (complete and valid cases, n=52).

Food category	% Total expenditures				% Total number of items			
	Means		Difference estimate	*P*	Means		Difference estimate	*P*
	Virtual estimate^a^	Real estimate			Virtual estimate	Real estimate		
Alcoholic beverages	5.15	3.47	1.68	.13	1.96	1.46	0.50	.43
Baby care	0.83	0.53	0.30	.43	1.21	0.96	0.26	.54
Bread and bakery	10.0	8.15	1.89	.02	11.7	10.3	1.44	.08
Baking and cooking	1.10	0.56	0.54	.16	0.98	0.85	0.39	.17
Beverages	5.03	5.92	–0.89	.30	4.63	5.32	–0.69	.21
Cereal and cereal products	5.64	7.14	–1.50	.06	5.46	7.58	–2.13	.01
Convenience foods	1.70	3.07	–1.38	.10	1.68	3.35	–1.70	.02
Dairy	19.1	12.6	6.49	<.001	13.6	11.4	2.21	.04
Deli and chilled foods	0.31	0.07	0.25	.15	0.28	0.07	0.21	.29
Edible oils and emulsions	3.28	3.16	0.12	.84	2.46	2.35	0.11	.81
Eggs	3.78	2.15	1.63	.01	2.94	1.50	1.44	.003
Fish and fish products	2.82	2.44	0.38	.42	2.88	2.65	0.23	.69
Fruit and vegetables fresh	14.3	17.4	–3.12	.04	28.9	22.9	5.94	.002
Fruit and vegetables other	5.46	7.36	–1.90	.009	6.57	8.64	–2.07	.02
Fresh meat, meat products, fresh fish	16.5	16.8	–0.31	.85	9.64	9.77	–0.13	.89
Other miscellaneous	0.05	0.42	–0.36	.07	0.07	0.35	–0.28	.12
Sauces, spreads, and seasonings	3.38	4.56	–1.18	.05	3.29	4.79	–1.50	.01
Snack foods	1.58	4.17	–2.59	<.001	1.83	5.88	–4.05	<.001

^a^ Results from repeated measures mixed models fitted to evaluate the percentage of price (outcome 1) and percentage of items (outcome 2), respectively, with supermarket type (virtual or real) and shop (1, 2, or 3) as fixed effects, registration number as random effect, and shop as repeated effect.

### Difference Between Virtual and Real Purchases

Table 3 shows the estimated means and difference for the virtual and real purchases for all 18 food categories and separately as a proportion of total expenditures (outcome 1) and proportion of total items (outcome 2) for the complete cases (n=52). Results for the total sample (N=86) are shown in Multimedia Appendix 1 and show comparable outcomes to the results presented subsequently. Mixed models revealed that the effect of shopping occasion (shop 1, 2, or 3) was only significant for the convenience foods category. This means that the similarity between virtual and real purchases was stable over time and, therefore, the results are presented at an aggregate level. Six food categories showed significant differences in purchases between the virtual and real shop, including bread and bakery (difference estimate 1.9%, *P*=.02); dairy (6.5%, *P*<.001); fruit and vegetables, fresh (3.1%, *P*=.04); fruit and vegetables, other (1.9%, *P*=.009); snack foods (2.6%, *P*<.001); and eggs (1.6%, *P*=.01). Similar results were observed for difference in proportion of items purchased.

### Focus and Presence

Previous research indicated that people with a higher focus might perceive stronger feelings of presence in virtual environments. Outcomes of the linear mixed models confirmed that focus was strongly positively associated with perceived presence in the Virtual Supermarket (estimate 1.05, *P*<.001).

With regard to presence, results showed that a large majority of participants (≥95% across the 3 shops) reported a medium-to-high feeling of presence in the Virtual Supermarket (Table 4). Participants scored particularly high for the subscales adaptation/immersion (ie, perceiving oneself to be enveloped by, included in, and interacting with an environment) and interface quality. According to theory, we would expect that people with a higher presence would perform better in the Virtual Supermarket compared to people with a lower perception of presence. Sensitivity analysis on a subset of participants with a high presence score (n=25) revealed greater similarity in proportional expenditures between virtual and real purchasing behavior. The total number of categories with significant differences between virtual and real purchases reduced from 6 to 4 in this subset of participants (the categories “fruit and vegetables” and “eggs” showed no longer significant differences).

**Table 4:** Mean total presence score^a^ and subscales for all participants who completed at least 1 virtual shopping occasion (n=83^b^).

Presence score and subscales for 3 groups		Mean (SD)	n (%)
**Total presence (range 29-203)**		104 (22.0)	
	Low (0-67)		3 (4)
	Medium (68-134)		73 (88)
	High (≥134)		7 (8)
**Subscale 1: involvement (range 12-84)**		40.0 (11.1)	
	Low (0-28)		12 (15)
	Medium (29-56)		67 (81)
	High (≥57)		4 (5)
**Subscale 2: sensory fidelity (range 6-42)**		18.0 (4.8)	
	Low (0-14)		19 (23)
	Medium (15-28)		62 (75)
	High (≥29)		2 (2)
**Subscale 3: adaptation/immersion (range 8-56)**		31.2 (6.9)	
	Low (0-18)		1 (1)
	Medium (19-37)		64 (77)
	High (≥38)		18 (22)
**Subscale 4: interface quality (range 3-21)**		14.9 (3.2)	
	Low (0-7)		1 (1)
	Medium (8-14)		33 (40)
	High (≥15)		49 (59)
**Focus (range 7-49)**		29.7 (5.7)	
	Low (0-16)		—
	Medium (17-32)		51 (61)
	High (≥33)		32 (39)

^a^ Mean represents the typical value for each participant over all recorded virtual shops.

^b^ Questionnaire data for 3 participants were missing due to technical issues.

## Discussion

This study shows that consumer purchasing patterns in the Virtual Supermarket were comparable to those in real supermarkets and confirms that the Virtual Supermarket is a valid tool to measure food purchasing behavior in a supermarket setting. Furthermore, a large majority of participants experienced a medium-to-high sense of presence in the Virtual Supermarket. The categories “fruits and vegetables” and “dairy” showed the strongest difference between virtual and real purchases. Results will be useful to guide further improvement of the software.

This validation study examined differences in purchasing patterns between virtual and real shopping occasions across a total of 18 different food categories. Results demonstrate that the 4 food groups with the highest relative expenditures were the same for the virtual and real shopping occasions (eg, fresh fruit and vegetables, bread and bakery, dairy, and meat and fish). Likewise, the 4 food groups with the lowest proportional expenditures were the same (eg, other miscellaneous, deli and chilled foods, baking and cooking goods, and baby care products). These results indicate that the overall purchasing patterns were similar in the virtual and real supermarket and that proportional purchases within food groups were comparable.

One key limitation of the Virtual Supermarket is that participants did not use real money and, therefore, could spend more than they would in real life. For this reason, participants were asked to set a realistic shopping budget and the software did not allow them to overspend. Results showed no patterns of overspending in the Virtual Supermarket compared to the real supermarket; for some food categories, people did spend more in the Virtual Supermarket (dairy, bread and bakery, and eggs) but for others they spent less (fruit and vegetables, and snack foods). Unexpectedly, total absolute expenditures were substantially lower in the Virtual Supermarket compared to the real supermarket. On average, participants spent approximately 70% of their shopping budget in the Virtual Supermarket (which is reasonable); however, in approximately 10% of the shopping occasions, participants spent less than 30% of their budget. One plausible explanation for the relatively low expenditures is that the Virtual Supermarket holds no sales promotions. A 2002 study by Aguirregabiria [28] revealed that typical sales promotions (which are approximately a 20% discount) increase weekly sales rates by 500%. In addition, choices are often influenced by other in-store promotions, such as end-of-aisle and merchandising displays [29], none of which are available in our Virtual Supermarket.

We could include promotions in future versions of the Virtual Supermarket; however, when designing RCTs it is important to realize that these features might influence the outcome of interest. One key advantage of the Virtual Supermarket compared to real supermarkets is that it enables creation of a “neutral” shopping environment with completely controllable experimental and control conditions enabling effective testing of the effects of changing only one element (eg, price) without interference of other effects (eg, sales signs). Also, it can be used nationwide because it does not require participants to come to a specific research location. Depending on the specific study aims, it is important to consider the balance between making the Virtual Supermarket as realistic as possible (external validity) and designing it to deliver a valid study outcome (internal validity). A major advantage of the Virtual Supermarket is that it is modifiable and researchers can design the Virtual Supermarket in accordance with specific study needs.

In addition to overall shopping patterns, this study compared proportional purchases within 18 food categories. Results demonstrated that 6 of the food categories differed significantly in proportion of total expenditures between the virtual and real shops. These categories were bread and bakery; dairy; fruit and vegetables, fresh; fruit and vegetables, other; snack foods; and eggs. Although this finding is important, it needs to be interpreted with some caution. Most importantly, we made multiple comparisons between groups, which increased the likeliness of finding significant results. A more conservative level of significance of *P=*.01 might be appropriate, in which case only 3 categories (dairy, processed fruit and vegetables, and snack foods) would have shown relevant significant differences. Furthermore, our focus was on proportional purchases, meaning that if one food category showed clear differences, other food categories would be affected as well (eg, if participants spent 17% on meat in the Virtual Supermarket there was only 83% left for other categories). The reason why we focused on relative purchases and not absolute purchases is that the prices in the Virtual Supermarket and the real supermarket were expected to be different due to promotional and seasonality effects in the real supermarket. Finally, it is important to consider that our findings are quite conservative. Our conclusions are based on comparing shopping behavior in 2 different environments. However, it also can be expected that 2 shops in the same environment can be slightly different (eg, people do not buy exactly the same each time). Our analysis did not account for this “natural” variation in shopping behavior, which further strengthens our finding that the differences between the Virtual Supermarket and the real supermarket were relatively minor.

Keeping this in mind, food categories that showed the most important differences between the virtual and real shopping occasions were fresh fruit and vegetables, dairy, and snack foods. For fresh fruit and vegetables, participants spent significantly more in real life compared to the virtual shops. One important explanation for this finding is that many participants misunderstood the interface in the Virtual Supermarket for purchasing loose items. After clicking on a fruit or vegetable product, participants were asked to enter the number of grams they wanted to buy (eg, 100 grams of apples). However, we observed that a large number of participants (n=29) misinterpreted this question and entered the number of items they wanted to buy (eg, 2 apples). The software registered this automatically as grams, meaning that the till receipt would show 2 grams of apples. This error caused a significant underrepresentation of fruit and vegetable expenditures in the Virtual Supermarket and if these participants were excluded from analysis the proportional expenditures on fruit and vegetables increased from mean of approximately 14% (SD 14) to a mean of approximately 15% (SD 15). This effect might also partly explain the observed underspending in the Virtual Supermarket as mentioned previously. Therefore, improving the interface for loose items will be a key priority when updating the Virtual Supermarket. Looking at snack foods, the relative underspending within this category in the Virtual Supermarket could be due to a number of reasons. First, snack foods are normally heavily promoted which leads to increased sales [29-31]. This effect was not captured in the Virtual Supermarket. Also, supermarkets tend to provide more shelf space for snacks foods, which is expected to increase sales [32]. The Virtual Supermarket used a representative product selection based on the number of different types of products/brands available for sale in each food category in NZ (eg, different types of soft drinks), not accounting for the exact number of individual products that is normally displayed on the shelves (eg, supermarkets often display a large number of the same Coca-Cola bottles covering a whole aisle; the Virtual Supermarket did not account for this). To improve the resemblance with real supermarkets, it might be important to measure shelf space for all products in real supermarkets and design the Virtual Supermarket accordingly. However, there is a large variation in design between different supermarkets making it hard to determine the best representation; also, this requires cooperation from supermarkets which might be difficult to achieve.

Another limitation of this study was the relatively large number of participants that dropped out due to software problems. In particular, a large number of participants reported that the software could not be run on their (mostly older) computers. Since completion of this validation study, we have found a solution to this by improving the way the software processes the individual product images. Therefore, for future studies, the NZ Virtual Supermarket will be more compatible with older computers and laptops. Nevertheless, the high dropout could bias our results and participants that completed the study might have been better using the software than an average user. Another problem was that we were not able to recruit an ethnically diverse sample and, therefore, are not able to draw conclusions on the acceptability and usability of the Virtual Supermarket among Māori (indigenous) and Pacific people. To ensure diversity in future studies, it would be important to use targeted recruitment strategies for these populations. Nevertheless, our sample was relatively diverse with regard to other characteristics, including education level, age, work status, and income, and there were no signs of differential dropout.

To our knowledge, this is one of the first studies making a direct comparison between virtual and real shopping behavior. One other example is a 2004 study by Grewe et al [33] who validated a virtual reality (VR) shopping task to assess real-life memory and spatial navigation in patients with epilepsy. The authors found that the VR tool was valid and generalizable compared to a real-life shopping task. Furthermore, Sharpe et al [34] validated fast food and soft drink purchases made in an online simulated road trip against actual purchasing and consumption of a lunch meal from McDonalds 1 week later. This study found strong evidence that virtual and real purchases were similar and that participants were consistent in the type of foods they chose in the virtual task [34]. In other health-related domains, there is more evidence for the realism and performance of virtual environments. For example, VR is frequently used to improve surgical technical skills [35], as a therapeutic instrument in clinical psychology [36,37], and in the treatment of several phobias [38]. Also, in the field of computer sciences, there is a wealth of research into human computer interaction [39]. Linking this evidence to the Virtual Supermarket, there is good reason to assume that people experience a high presence because of the high realism (eg, it requires very little imagination to recognize the software as a supermarket), the realistic movement (pushing a shopping trolley, 3D features), and the fact that it is easy to locate oneself due to clear landmarks (eg, aisles) [39]. One way to further improve presence is through the use of avatars. People tend to feel a higher feeling of presence when there is a virtual representation of oneself in the virtual world and when other users recognize them (other avatars) [39]. It would be interesting to explore the use of avatars further and to test the impact of social interactions in the Virtual Supermarket.

Another way to improve the feeling of presence is by improving software graphics and functionality. With improving technology, virtual environments are becoming increasingly realistic and we aim to improve the Virtual Supermarket alongside improving technology. Although we realize that virtual worlds will never be the same as real life, they do provide a clear opportunity to conduct research that would otherwise be nearly impossible. In addition, the Virtual Supermarket offers the opportunity to provide much needed independent rigorous experimental evidence to inform (controversial) food policy [40].

This study shows that the Virtual Supermarket is a valid and useful tool to study food-purchasing behavior. The results of this validation will provide valuable guidance regarding further improvements to the software and to the optimization of the internal and external validity of this innovative methodology.

## Multimedia Appendix 1

Means and difference for Virtual Supermarket and real supermarket purchases for the 18 food categories (total sample, n=86).

## Abbreviations

RCT: randomized controlled trials
SMS: short message service
VR: virtual reality
