# Supplementary material for: Using a 3D Virtual Supermarket to Measure Food Purchase Behavior: A Validation Study
Source: J Med Internet Res. 2015 Apr 28;17(4):e107. doi: 10.2196/jmir.3774 (PMC4429224; doi:10.2196/jmir.3774)
Supplement: Supplementary file 1 [file jmir_v17i4e107_app1.pdf]

APPENDIX 1 Means and difference for Virtual Supermarket and real supermarket purchases for the 18 food categories (total sample, n = 86)

Food category	% Total expenditures				% Total number of items			
	Means		Differen ceEstim at e	*P* - value	Means		Differen ce Estimate	*P* - value
	Virtual Estimate [a]	Real Estimate			Virtual Estimate	Real Estimate		
Alcoholic beverages	4.06	3.36	0.70	.46	1.60	1.47	0.14	.80
Baby care	0.86	0.45	0.24	.45	0.98	0.85	0.13	.72
Bread and bakery	10.8	8.42	**2.35**	**.003**	12.7	10.9	**1.80**	**.03**
Baking and cooking	0.95	0.51	0.44	.15	0.93	0.57	0.36	.11
Beverages	5.04	5.97	-0.93	.21	4.78	5.48	-0.71	.18
Cereal and cereal products	5.91	6.95	-1.04	.16	5.88	7.32	**-1.44**	**.049**
Convenience foods	1.89	2.87	-0.97	.18	1.84	3.22	**-1.38**	**.02**
Dairy	18.5	12.2	**6.36**	**<.001**	13.1	11.3	1.83	.05
Deli and chilled foods	0.22	0.04	0.18	.18	0.21	0.06	0.15	.32
Edible oils and emulsions	3.04	3.15	-0.11	.82	2.43	2.45	-0.01	.97
Eggs	3.60	2.00	**1.60**	**.003**	2.84	1.41	**1.43**	**<.001**
Fish and fish products	2.94	2.58	0.36	.44	2.72	2.56	0.16	.74
Fruit and vegetables fresh	13.5	19.8	**-3.29**	**.02**	27.2	21.7	**5.51**	**.001**
Fruit and vegetables other	5.95	7.58	**-1.63**	**.02**	7.03	8.92	**-1.89**	**.02**
Fresh meat, meat products, fresh fish	17.1	17.5	-0.38	.81	9.92	10.2	-0.32	.74
Other miscellaneous	0.17	0.40	-0.23	.20	0.13	0.35	-0.23	.12
Sauces, spreads, and seasonings	3.78	4.96	**-1.8**	**.03**	3.55	5.13	**-1.58**	**.002**
Snack foods	1.85	4.06	**-2.21**	**<.001**	2.08	5.65	**-3.57**	**<.001**

[a] Results from repeated measures mixed models fitted to evaluate the percentage of price (outcome 1) and percentage of items (outcome 2), respectively, with supermarket type (virtual or real) and shop (1,2 or 3) as fixed effects, registration number as random effect and shop as repeated effect.
